# Internal consistency and factor structure of a brief scale assessing sensitivity to blood, injury, and mutilation

**DOI:** 10.1186/s13104-019-4200-9

**Published:** 2019-03-25

**Authors:** Oscar I. Gonzalez, James A. Naifeh, Quinn M. Biggs, Tsz Hin Hinz Ng, Carol S. Fullerton, Robert J. Ursano

**Affiliations:** Center for the Study of Traumatic Stress, Department of Psychiatry, Uniformed Services University of the Health Sciences, 4301 Jones Bridge Road, Bethesda, MD 20814 USA

**Keywords:** Assessment, Mental health, Sensitivity to blood, injury and mutilation (SBIM), Mutilation Questionnaire (MQ), Post-traumatic stress disorder (PTSD), Risk factor, Soldiers

## Abstract

**Objective:**

US Army soldiers and military veterans experience high rates of post-traumatic stress disorder (PTSD). However, PTSD risk factors are not fully understood. Sensitivity to blood, injury, and mutilation (SBIM), which includes fear of being injured, seeing another person injured, and exposure to mutilation-relevant stimuli (e.g., blood, wounds) may be a PTSD risk factor that is identifiable prior to trauma exposure. Building on previous research that used a subset of items from the Mutilation Questionnaire (MQ), the aim of this study was to examine the reliability and validity of two brief scales assessing SBIM.

**Results:**

Data from two independent samples of male, US Army soldiers, was utilized to examine a brief 10-item SBIM measure (MQ-SBIM-10) and a shorter version 5-item SBIM measure (MQ-SBIM-5). Internal consistency was indexed by the Kuder–Richardson 20 formula. Construct validity was assessed using confirmatory factor analysis and results obtained from each sample, and from a combined sample. The MQ-SBIM-10 demonstrated acceptable internal consistency and the hypothesized one-factor structure. Although the MQ-SBIM-5 explained a substantial amount of the variance in the 10-item measure and had a one-factor structure, internal consistency of the 5-item measure was poor. Analyses supported the MQ-SBIM-10 as a reliable and cohesive measure of sensitivity to blood, injury, and mutilation.

## Introduction

Rates of post-traumatic stress disorder (PTSD) are high among US Army soldiers and military veterans [1, 2]. Risk factors for developing PTSD are not fully understood. Sensitivity to blood, injury, and mutilation (SBIM), which includes fear of being injured, seeing another person injured, and exposure to mutilation-relevant stimuli (e.g., blood, wounds) may be a PTSD risk factor that is identifiable prior to trauma exposure. Evidence suggests that peritraumatic responses may play an important role in the subsequent development of PTSD [3]. Exposure to injury and mutilation is associated with physiological and behavioral reactivity, as well as self-reported arousal, negative affect, and disgust [4–9]. Considering that traumatic events often involve actual or perceived threat of injury to oneself and others, individuals with heightened sensitivity to these stimuli may experience elevated psychological and/or physiological responses that increase risk for post-traumatic stress.

PTSD symptoms of intrusion and avoidance have been positively associated with fear of gruesome experiences and potential bodily injury [9] as measured by the 30-item Mutilation Questionnaire (MQ) [10]. First reported by Klorman et al. [10], the MQ is a self-report inventory designed to assess the cognitive-verbal component of fear of gruesome tasks or events where bodily injury is possible. However, an exploratory factor analysis (EFA) of the MQ using college student data revealed a heterogeneous factor structure suggesting that the MQ may not be a cohesive measure of a unitary construct [11]. A recent EFA of the MQ using data from US soldiers similarly found a heterogeneous factor structure [12]. Importantly, a subset of 10 MQ items was identified that form a cohesive SBIM scale focused on sensitivity to injury (to oneself and others) and mutilation-related stimuli that may accompany injuries (e.g., blood, wounds) [12]. The sum of those 10 MQ items (called here MQ-SBIM-10) was positively associated with PTSD symptom severity, even after controlling for well-established demographic risk factors (age, education, military rank), lifetime trauma exposure, and trait neuroticism [12].

The degree to which SBIM reflects a state-like or trait-like construct remains unclear. Animal and human studies suggest that fear of injury and mutilation may be a fundamental psychobiological phenomenon with implications for understanding anxiety-related disorders [13, 14]. While psychophysiological assessment of SBIM may be optimal, it is impractical for large-scale administration. Valid and reliable measurement is vital for improving our understanding of SBIM and its potential relationships with trauma exposure, PTSD, and other mental disorders.

Continued use of the MQ-SBIM-10 requires a more extensive examination of its psychometric properties. Early identification of SBIM may be particularly important in military populations where threat of injury and mutilation-related stimuli are intrinsic to combat and other military operations. However, given the challenges of large scale military screening assessments where brevity is critical to inclusion, it would be advantageous to shorten the MQ-SBIM-10 even further. To these ends, we conducted a series of analyses using data from two independent samples of male US Army soldiers, Special Operations Command (SOC) and Mortuary Affairs (MA). SOC soldiers are organized, equipped, and trained to conduct unconventional, high-risk, high-value combat operations where exposure to injury and death are likely. MA soldiers recover, identify, and evacuate the remains of the dead from the theater of war, duties that expose them to dismembered, burned, and decomposed remains and potential personal injury in the combat environment. We first investigated the internal consistency and construct validity of the MQ-SBIM-10 in both samples. Construct validity was assessed using confirmatory factor analysis (CFA), a more rigorous test than the EFA reported in previous research [12]. We then repeated these analyses using a 5-item subset of the MQ-SBIM-10.

## Main text

### Methods

De-identified, cross-sectional data were obtained from 675 SOC and 750 MA soldiers, all males. All soldiers completed a self-report questionnaire as part of their voluntary participation in one of two larger studies on mental health outcomes in military personnel. Data was collected between 2009 and 2011 for SOC soldiers [12], and between 2005 and 2015 for MA soldiers. Study participants provided written informed consent. Both studies were approved by the Institutional Review Board at the Uniformed Services University of the Health Sciences, Bethesda, MD.

### Measures

Demographic and military variables included age, race/ethnicity (White non-Hispanic vs. other), marital status (married vs. single), education (high school or less vs. more than high school), rank (E1–E4 vs. E5 or higher), previous deployment to the Middle East region (yes vs. no). Lifetime combat exposure (e.g., *Being attacked; Handling or uncovering remains*) was based on the sum of 27 items (never = 0 and yes = 1) adapted from the Combat Experiences Scale (CES) [15]. The CES had excellent internal consistency in both samples (SOC, α = 0.95, *n* = 675; MA, α = 0.92, *n* = 284).

SBIM was assessed using the subset of 10 MQ items (MQ-SBIM-10; see Table 1) previously identified by Naifeh et al. [12]. Items (e.g. *“Open wounds nauseate me”*) are endorsed true = 1 or false = 0 and summed to generate a SBIM severity score. A 5-item version (MQ-SBIM-5) was also generated by retaining items with the highest item-total correlations among both study samples (data available upon request) while excluding items with convoluted wording or highly overlapping content (Table 1).

**Table 1:** Item content for the MQ-SBIM-10 and MQ-SBIM-5

Sharp knives make me nervous^a^
Cuts and wounds upset me
Open wounds nauseate me^a^
Injuries, accidents, blood bother me^a^
Turn away from badly injured person on TV
I dislike looking at pictures of accidents^a^
Power tools make me nervous
Feel faint if I saw a wounded eye^a^
Shudder when I think of cutting myself
Frightened I might have to help an injured person

Items in the table are abbreviated/paraphrased

^a^Item included in the MQ-SBIM-5

### Statistical analyses

Between-sample differences on categorical and continuous variables were examined using Chi square tests and unpaired *t*-tests, respectively. Internal consistency of the MQ-SBIM-10 and MQ-SBIM-5 was examined using the Kuder–Richardson 20 formula (KR-20), which is appropriate for scales with dichotomous items [16], and inter-item correlations. To examine construct validity, we used confirmatory factor analysis (CFA) to estimate the hypothesized one-factor structure of each SBIM scale.

We also performed a multi-group CFA where factor loadings were held constant across the SOC and MA samples (n = 1425). In accordance with recommendations [17], the following CFA fit indices were examined: the Tucker–Lewis fit index (TLI; > 0.90 = acceptable, > 0.95 = excellent), comparative fit index (CFI; > 0.90 = adequate), RMSEA (< 0.05 = good, 0.05–0.08 = adequate, 0.08–0.10 = marginal, > 0.10 = poor), and Chi square test of model fit. Finally, linear regression analyses examined the proportion of MQ-SBIM-10 total score variance explained by MQ-SBIM-5 total scores in each sample. Analyses were conducted using SAS 9.4 [18] and MPlus [19].

### Results

#### Sample

Compared to the MA, the SOC were older (29.9 vs 26.9 years) and more likely to report White non-hispanic race/ethnicity (66.8% vs 43.5%), more than high school education (73.0% vs 61.3%), being currently married (65.9% vs 53.1%), rank E5 or higher (70% vs 30%), previous deployment to the Middle East (100% vs 37.9%), and more combat exposure, *t* = 4.89 (669), *p* < 0.0001 (Table 2). The samples did not differ on MQ-SBIM-10 total score, but the MQ-SBIM-5 total score was significantly higher for SOC, *t* = 2.36 (1423), *p* = 0.0182 (Table 2).

**Table 2:** Sample characteristics and descriptive statistics of study variables

Variable	SOC (*n* = 675)	MA (*n* = 750)	*𝜒* ^2^	*df*	*p* value
	% (*n*)	% (*n*)			
*Ethnicity*			78.1	1	< 0.0001
White non-hispanic	66.8% (451)	43.5% (326)			
Other	33.2% (224)	56.5% (424)			
*Education*			22.0	1	< 0.0001
≤ High school	27.0% (182)	38.7% (290)			
> High school	73.0% (493)	61.3% (460)			
*Marital status*			24.3	1	< 0.0001
Married	65.9% (445)	53.1% (398)			
Single	34.1% (230)	46.9% (352)			
*Rank*			230.0	1	< 0.0001
E1-E4	30.2% (204)	70.4% (528)			
≥ E5	69.8% (471)	29.6% (222)			
*Deployment* ^a^			623.2	1	< 0.0001
No	0.0%	62.1% (466)			
Yes	100.0% (675)	37.9% (284)			

	*M* (SD)	*M* (SD)	*t*	*df*	*p* value
Age in years	29.9 (7.3)	26.9 (7.2)	7.80	1423	< 0.0001
CES^b^	12.5 (8.3)	10.0 (6.6)	4.89	669	< 0.0001
MQ-SBIM-10	0.9 (1.5)	0.8 (1.5)	1.83	1423	0.0670
MQ-SBIM-5	0.5 (0.9)	0.4 (0.9)	2.36	1423	0.0182

^a^Previous deployment to the Middle East region

^b^CES data was examined for those who had previously deployed to the Middle East region (*n* = 675 for SOC; *n* = 284 for MA)

### Internal consistency and construct validity

#### MQ-SBIM-10

The MQ-SBIM-10 demonstrated acceptable internal consistency in both samples, with KR-20 of 0.72 and 0.78 among SOC and MA, respectively. Item-total correlations ranged from 0.29 to 0.50 for the SOC and from 0.34 to 0.56 for the MA. Average item-total correlations were 0.40 for SOC and 0.45 for MA, exceeding the acceptable lower limit of 0.30 [20].

The CFA results confirmed that the one-factor model proposed for the 10-item SBIM index fit the data reasonably well (Table 3). The CFI and the TLI were at or above the 0.90 standard for acceptable fit in both the SOC and MA groups, and the RMSEAs were all smaller than 0.06, indicating acceptable/good fit of the model hypothesized [17]. The CFA indices obtained from the combined group also supported the one-factor structure (i.e., RMSEA ≤ 0.06, CFI ≥ 0.95, TLI ≥ 0.95) [17].

**Table 3:** Properties of SBIM factor structures among SOC (n = 675), MA (n = 750), and combined sample (n = 1425)

Model	*𝜒* ^2^	*df*	P	RMSEA (90% CI)	CFI	TLI
*10-item SBIM factor*						
SOC	117.965	35	< 0.0001	0.059 (0.048–0.071)	0.922	0.900
MA	71.515	35	0.0003	0.037 (0.025–0.050)	0.976	0.969
Combined	186.939	78	< 0.0001	0.044 (0.036–0.052)	0.958	0.952
*5-item SBIM factor*						
SOC	6.298	5	0.2783	0.020 (0.000–0.060)	0.997	0.993
MA	1.340	5	0.9308	0.000 (0.000–0.015)	1.000	1.015
Combined	9.374	13	0.7441	0.000 (0.000–0.027)	1.000	1.006

#### MQ-SBIM-5

The reliability coefficients (KR-20) for MQ-SBIM-5 were 0.57 and 0.67 among SOC and MA, respectively. Item-total correlations ranged from 0.22 to 0.45 in SOC, and from 0.38 to 0.48 in MA. Average item-total correlations were 0.36 for SOC and 0.43 for MA. Results also supported a single-factor conceptualization of this measure as CFA fit indexes met the recommended cut-offs for good fit in all three models (CFI and TLI exceeded 0.95 and RMSEA was below 0.06). A linear regression analysis indicated that MQ-SBIM-5 sum scores explains 82% and 85% of variation in MQ-SBIM-10 scores in the SOC and the MA, respectively.

### Discussion

The current study provided initial examination of the reliability and validity of two brief measures of SBIM, a potential risk factor for PTSD. Building on previous research [12], the MQ-SBIM-10 (10 items) and MQ-SBIM-5 (5 items) were examined using data from two independent samples of male, US Army soldiers. Between-group differences in demographic and military variables were not surprising given the nature of these units’ occupations. The average MQ-SBIM-10 score did not significantly differ between these groups, which could suggest that SBIM may be a trait-like construct. However, mean scores were significantly higher among SOC compared to MA for the MQ-SBIM-5. Additional research is needed to begin to elucidate the nature (state-like or trait-like) of the SBIM construct.

The MQ-SBIM-10 demonstrated acceptable internal consistency (KR-20 = 0.72). Internal consistency of the MQ-SBIM-5 was low (KR-20 = 0.57). We expected some reduction in reliability due to fewer scale items. However, the poor internal consistency of the MQ-SBIM-5 limits its current utility, points to the need for further examination, and suggests that researchers should use the MQ-SBIM-10 at the moment. Inter-item correlations were positive, and none was high enough for any item to be redundant. In both samples, the internal consistency coefficients (KR-20) were larger for the MQ-SBIM-10 than for the MQ-SBIM-5, which was also expected given that such values tend to be lower with fewer items [21].

The hypothesized one-factor structure of the MQ-SBIM-10 and MQ-SBIM-5 was supported by the CFA results obtained from the SOC, MA, and the combined group. It may be argued that unidimensional measures, by virtue of containing only items of the construct of interest, promote parsimony of scale items which reduces questionnaire length and respondent fatigue.

## Limitations

Due to the use of cross-sectional data and convenience sampling, generalizability is limited and test–retest reliability was not assessed. Analyses were based on existing data sets, hence, the MQ-SBIM-10 and MQ-SBIM-5 were not administered independent of each other or the full MQ, and results could have varied if administered separately. Further investigation of the reliability and validity of the proposed measures is warranted.

## Abbreviations

PTSD: post-traumatic stress disorder
SBIM: sensitivity to blood, injury, and mutilation
MQ: Mutilation Questionnaire
EFA: exploratory factor analysis (EFA)
SOC: special operations command (SOC)
MA: mortuary affairs (MA)
CFA: confirmatory factor analysis (CFA)
CES: Combat Experiences Scale (CES)
MQ-SBIM-5: 5-item measure of sensitivity to blood, injury, and mutilation
MQ-SBIM-10: 10-item measure of sensitivity to blood, injury, and mutilation
RMSEA: root mean square error of approximation
SAS: statistical analysis system
CFI: comparative fit index
TLI: Tucker Lewis Index
